# Introduction and Tribute to Charlie Calisher

**DOI:** 10.3390/diseases9040075

**Published:** 2021-10-20

**Authors:** Thomas P. Monath, Frederick A. Murphy

**Affiliations:** 1 Vaxxinity, Dallas, TX 75201, USA; 2 School of Veterinary Medicine, University of California, Davis, CA 95616, USA; famurphy@utmb.edu; 3 Medical Branch, University of Texas Medical Branch, Galveston, TX 77550, USA

It is a great pleasure to contribute a few words of introduction to this Special Issue of MDPI’s *Diseases* entitled “Recent Studies of Arthropod-, Bat-, and Rodent-Borne Viruses: A Theme Issue in Honor of Professor Charles H. Calisher”. Charlie has been our close friend and colleague for more than 55 years. We have also known almost all the pioneers who built the discipline of arbovirology going back to days of the Rockefeller Foundation Virus Program. This facet of tropical medicine and public health attracted some of the most creative and unique characters in memory, characters larger than life, characters who had a firm handle on the complex interaction between the viruses, the hosts and the natural world—they were and are a community of scholars with a holistic, global view and a joie de vivre. Charlie, from day one, became an activist in this community and soon dug out a unique, pioneering niche within it. These were heady times in arbovirology, in the field, in the lab, in public health practice.

Charlie was recruited in 1965 by the legendary Rockefeller Foundation virologist Telford Work to the then Arbovirus Infections Unit, Viral Diseases Section, Communicable Disease Center, Atlanta, Georgia (there would be many unit name changes in the succeeding years, always with the same abbreviation: CDC). It was at the CDC where we had the pleasure to meet and then work with Charlie over so many good years. This continued, of course, from 1993 onward when Charlie was appointed professor of microbiology at Colorado State University, and the beat goes on…

Charlie has written that his special interests include “the evolution of arboviruses, rodent-borne viruses, bat-borne, and other viruses; the ecology, epidemiology, and epizoology of viruses; virus classification and taxonomy; and the development of rapid methods for arbovirus and rodent-borne virus detection and identification”—big chunks, all key building blocks of today’s arbovirology. One measure of his success in all this is his record of >300 publications, >75 book chapters and reviews, and 18 books—not to mention numerous op-eds and published commentaries. Charlie has named far more new viruses than any living member of the arbovirology community and, if given the opportunity, will relate an interesting vignette about each of them. He has also helped to construct the complex taxonomy of the taxa containing the arboviruses and has been a devoted member of the ICTV. A small reward for his taxonomic work was the naming of a new flea for him—*Castallagia calisheri*, picked from, appropriately, a *Peromyscus maniculatus* deer mouse, a species Charlie later studied for many years as a host of the sin nombre virus (an etiologic agent of hantavirus cardiopulmonary syndrome).

Charlie has been involved in the response to numerous arbovirus epidemics, not only with responsibility for serological and virological laboratory activities, but also with substantial field work (see Figure 1), where he has always exhibited a keen eye for the ecological circumstances involved and has helped to develop longitudinal follow-up studies on the underlying ecology. Charlie has always taken a broad interest in the work of colleagues in different disciplines, all necessary in understanding and combating viral disease outbreaks. He has always been seen at the interface between virology, ecology, animal biology, wildlife biology, ornithology, entomology, and the clinical medical and veterinary sciences. Another dimension of Charlie’s renaissance view is his ability to extend from the exceptional and arcane to the sweeping and seminal—for example, contrast his paper “Antibodies to arboviruses in sera of domestic animals on a Dalmatian island” (with Punda and others, Vet Archiv (1985;55:225)) with his works on the spread of viruses by migratory birds, the antigenic classification of the member viruses of all the taxa that include arthropod-borne and rodent-borne members, the role of deer mice in hantavirus epidemiology, and the importance of bats as reservoir hosts of medically important viruses. Moreover, he has always been seen at the interface between our domestic arbovirus problems and their global parallels. He lists in his CV, under “Countries worked in (one week to one year)”, 41 countries! Much of the grand adventure of all this is captured in a personal way by Charlie in his classic history book, *Lifting the impenetrable veil: From yellow fever to Ebola hemorrhagic fever and SARS* (Rockpile Press, Ft Collins, CO, USA, 2013).

Lastly, but perhaps most important, is Charlie’s human side. We know of the primacy in his life of his grand family, and we know of his appreciation of his many lifelong friends. We know how this links to his professional life—blessed with a gift of intelligence and a phenomenal memory, a creative personality, a disarming wit and sense of humor, and a gift of gab worthy of an Irish poet, Charlie has not only been a thought leader but a devoted mentor and reliable friend to students and colleagues around the world. There are so many ‘Charlie stories’ that it is difficult to select one as an example of his unique character. Perhaps the one that best illustrates his great-hearted character is the one about his trip to Cuba during the Castro era. Charlie was in a throng of people on a street when everyone looked up to see flames coming from windows high above and people screaming for help. Without hesitation Charlie ran into the building’s doorway, only to be escorted out by guards a few moments later—the event had been staged as a demonstration by the fire department, whose ladder trucks arrived on the scene moments later.

It is not surprising that Charlie has also contributed to civic causes and the education of children. His love of baseball is legendary (he was a minor league player before deciding to become a scientist)—he even went to umpire school in North Carolina, and for many years was coordinator of umpires for Fort Collins youth baseball.

Charlie has been awarded multiple honors over the course of his career, including the Richard Moreland Taylor Award from the American Committee on Arthropod-Borne Viruses (ACAV). It is certainly a befitting tribute to add this honor to Charlie’s CV, this volume in his name with contributed papers reflecting the ongoing progress of the science he so loves.

## Figures and Tables

**Figure 1:**
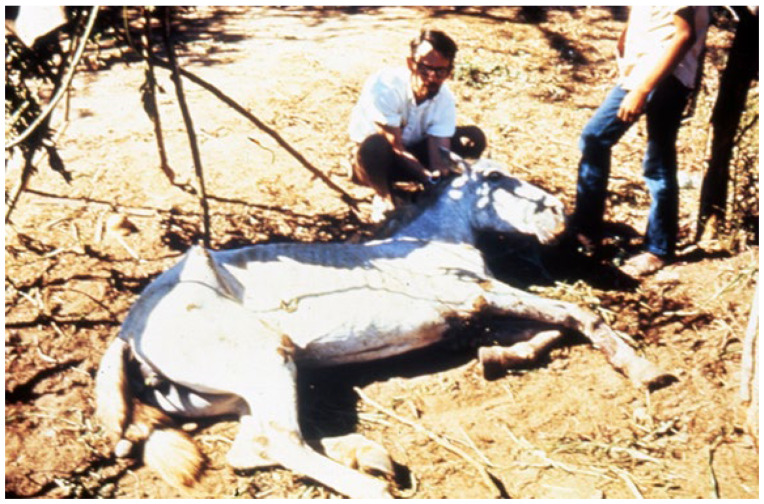
Charlie Calisher examining a horse infected with (and dying from) Venezuelan equine encephalitis virus near Nyarit, western Mexico, 1972.

